# Intrinsic Flame-Retardant and Thermally Stable Epoxy Endowed by a Highly Efficient, Multifunctional Curing Agent

**DOI:** 10.3390/ma9121008

**Published:** 2016-12-12

**Authors:** Chunlei Dong, Alvianto Wirasaputra, Qinqin Luo, Shumei Liu, Yanchao Yuan, Jianqing Zhao, Yi Fu

**Affiliations:** 1 School of Materials Science and Engineering, South China University of Technology, Guangzhou 510640, China; dongchunlei110@126.com (C.D.); alvianto_wira@yahoo.com (A.W.); lq198516@126.com (Q.L.); msycyuan@scut.edu.cn (Y.Y.); 2 School of Chemistry and Chemical Engineering, Lingnan Normal University, Zhanjiang 524048, China; 3 Silverage Engineering Plastics (Dongguan) Co., Ltd., Dongguan 523187, China; alvianto.wirasaputra@gmail.com

**Keywords:** flame-retardant, epoxy resin, thermal stability, blowing-out effect, thermosets

## Abstract

It is difficult to realize flame retardancy of epoxy without suffering much detriment in thermal stability. To solve the problem, a super-efficient phosphorus-nitrogen-containing reactive-type flame retardant, 10-(hydroxy(4-hydroxyphenyl)methyl)-5,10-dihydrophenophosphazinine-10-oxide (HB-DPPA) is synthesized and characterized. When it is used as a co-curing agent of 4,4′-methylenedianiline (DDM) for curing diglycidyl ether of bisphenol A (DGEBA), the cured epoxy achieves UL-94 V-0 rating with the limiting oxygen index of 29.3%. In this case, the phosphorus content in the system is exceptionally low (0.18 wt %). To the best of our knowledge, it currently has the highest efficiency among similar epoxy systems. Such excellent flame retardancy originates from the exclusive chemical structure of the phenophosphazine moiety, in which the phosphorus element is stabilized by the two adjacent aromatic rings. The action in the condensed phase is enhanced and followed by pressurization of the pyrolytic gases that induces the blowing-out effect during combustion. The cone calorimeter result reveals the formation of a unique intumescent char structure with five discernible layers. Owing to the super-efficient flame retardancy and the rigid molecular structure of HB-DPPA, the flame-retardant epoxy acquires high thermal stability and its initial decomposition temperature only decreases by 4.6 °C as compared with the unmodified one.

## 1. Introduction

Epoxy has been widely used in many fields such as electronics, electrical industries, aerospace, transportation, and coating due to its insulation, high strength, ease of processing, corrosion resistance, and high adhesion characteristics [1]. However, flammability is one of the main drawbacks of epoxy-based products because they lack flame-retardant components, which greatly restrains their advanced applications [2].

To overcome this inherent weakness, various fire-retarding agents have been explored in the past decades [3,4,5], by either reactive-type [6] or additive-type strategies [7,8,9]. Comparatively, the former was studied more intensely as incorporation of flame retarding units into epoxy networks by chemical means would generate higher efficiency and lessen the deterioration in other properties of the material [8,10]. Many studies indicate that a brominated flame retardant possesses highly efficient flame retardancy through the mechanism in gaseous phase [3], although the hazardous nature of the pyrolysis products, such as HBr, polybrominated dibenzo-*p*-dioxins, and furans, has caused an environmental burden that prohibits its production and usage [11,12,13]. As alternative counterparts, a halogen-free flame retardant carrying both phosphorus and nitrogen elements could significantly enhance the flame retardancy via the synergistic effect between these two elements [14,15,16]. The phosphorus-containing compounds decompose in advance during combustion, and the resultant pyrophosphates or polyphosphates catalyze the dehydration of the surrounding polymer matrix, forming a phosphorus-rich char layer that behaves as a protective barrier to inhibit the diffusion of heat and gaseous products [17]. Moreover, such substances might further disintegrate into phosphorus-containing species and play a flame-retarding role in the gaseous phase by scavenging H• and OH• radicals, thus intercepting the combustion [18]. Meanwhile, the nitrogen-containing moieties mainly release inert nitrogen, ammonia, and nitrogen oxide gases as thermal degradation products, not only to dilute the concentration of oxygen and combustible gas in the gaseous phase, but also to facilitate the expansion of the char layers formed during the burning of epoxy [19]. The co-action of these two elements with different functions might sometimes be referred to as a particular category of flame retardant, viz. intumescent flame retardant. In such circumstances, the polymer matrix encounters a stimulative decomposition by the phosphorus-containing substances. The compact char layer is formed afterwards and is then intumesced with the aid of a gas source from the nitrogen-containing compounds to create a porous foamed interior structure that serves as a physical barrier to slow down heat and mass transfer between the gaseous and condensed phases [20,21,22].

It is well known that 9,10-dihydro-9-oxa-10-phosphaphenanthrene-10-oxide (DOPO) moiety confers great flame retardancy in diglycidyl ether of bisphenol A/4,4′-methylenedianiline (DGEBA/DDM) system, though over 0.5 wt % of phosphorus content has to be loaded to achieve a decent outcome [23,24,25]. As a consequence of its high loading, the thermal stability of flame-retardant epoxy is significantly decreased. In our pilot work, an organic compound containing unique combination of phosphorus and nitrogen elements in the phenophosphazine ring, 5,10-dihydro-phenophosphazine-10-oxide (DPPA) [26], was synthesized and used as the co-curing agent for DGEBA/DDM. It could endow the cured thermoset with higher flame retardancy, in which the phosphorus content is diminished effectively, although no further analysis of the mechanism of such a result was reported. Moreover, an interesting phenomenon was observed during the vertical burning test of the samples. That is, after ignition, the pyrolytic gases spurted out from the material surface to blow off the flame. The so-called blowing-out effect was observed in DGEBA/diamine system with polyhedral oligomeric silsesquioxane containing DOPO (DOPO-POSS) as the flame retardant [27,28]. However, merely UL 94 V-1 rating in maximum was attained, despite the attempt with varying contents of flame retardant.

Referring to the chemistry of the reaction of DOPO that utilizes the reactivity of its electrophilic P–H bond [19,23,29,30,31], in this work, a novel DPPA derivative (labeled HB-DPPA) was synthesized via the addition reaction between DPPA and 4-hydroxybenzaldehyde (HB). It was then used as a co-curing agent of DGEBA/DDM. In comparison with DPPA, a more rigid benzene ring is included in the chemical structure of HB-DPPA, so the additional carbon would favor the formation of a firm char layer and hence the improvement of flame retardancy and thermal stability. Accordingly, the mechanism involved was studied with the aid of a comprehensive analysis of the thermal degradation behavior of the flame-retardant moiety and the formation of a char layer by both momentary ignition in a vertical burning test and sustained external heat in a cone calorimeter.

## 2. Results and Discussion

### 2.1. Synthesis and Characterization

In principle, HB-DPPA is produced through electrophilic addition reaction. That is, the electrophilic P–H group in DPPA reacts with the nucleophilic C=O group in HB. As shown by the Fourier transform infrared (FTIR) spectra of DPPA, HB, and HB-DPPA (Figure S1), a few peaks disappear after the addition reaction between DPPA and HB, like the distinctive absorption peak at 2370 cm^−1^ for P–H stretching vibration in DPPA and 1670 cm^−1^ for C=O stretching vibration in HB. Meanwhile, a broad absorption appears at around 3229 cm^−1^ for O–H. All the results confirm the occurrence of an addition reaction between DPPA and HB.

**Figure 1:**
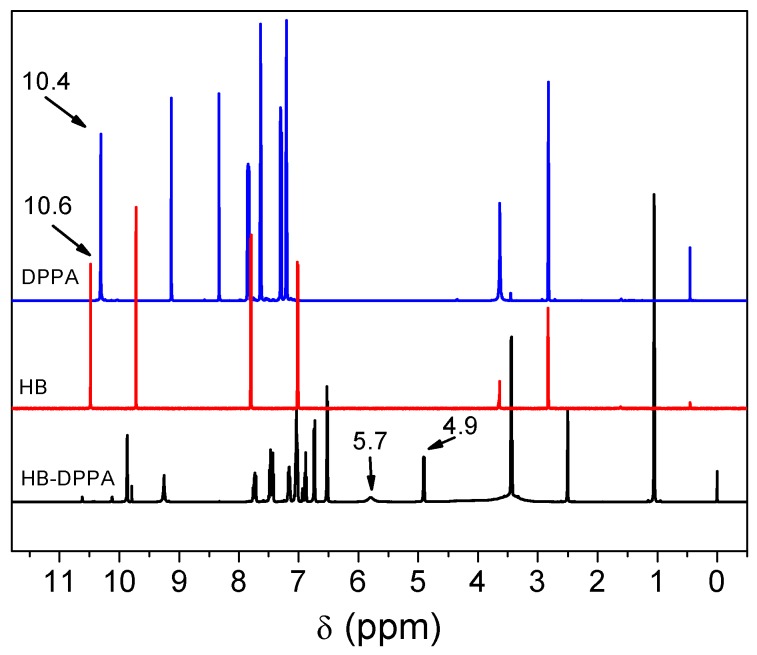
^1^H NMR spectra of DPPA, HB, and HB-DPPA.

Figure 1 shows ^1^H nuclear magnetic resonance (NMR) spectra of DPPA, HB, and HB-DPPA. The characteristic proton of C–OH at δ = 4.9 ppm is found in HB-DPPA, following the disappearance of the peak of P–H (δ = 10.4 ppm) in DPPA and the peak of C(O)–H (δ = 10.6 ppm) in HB. Moreover, the proton of –C(OH)–H in HB-DPPA is also observed at δ = 5.7 ppm.

Mass spectrometry (MS) m/z: calcd for C_19_H_16_NO_3_P: 337.31, found: 338.09 (M + H^+^), 360.07 (M + Na^+^).

Clearly, the product HB-DPPA with the expected chemical structure has been successfully synthesized.

### 2.2. Curing Behavior

The thermal curing behavior of the flame-retarded epoxy was investigated by differential scanning calorimeter (DSC) (Figure S2). The parameters of the curing process, including the onset curing temperature (T_o_), exothermic peak temperature (T_p_), and end curing temperature (T_e_), were derived from the thermograms. As shown in Table S1, the values of T_o_, T_p_, and T_e_ of EP-2 are all lower than those of EP-0. Since HB-DPPA is mixed first with DGEBA at high temperature until the solution becomes completely clear, it suggests that the N–H group in HB-DPPA molecule must have reacted with epoxide, producing a tertiary amine that catalyzes the subsequent curing reaction of DGEBA/DDM. As a consequence, the curing temperature decreases following the addition of HB-DPPA. The feasible reactivity of the N–H group in DPPA moiety with DGEBA is evidenced from the disappearance of the peak in ^1^H NMR analysis [26] and the appearance of the broad exothermic peak from 170 °C to 230 °C (Figure S3). Since the hydroxyl group possesses less nucleophilicity and would not attack the epoxide group in that temperature range, the detected exothermic peak represents the ring-opening reaction of DGEBA by the N–H group of HB-DPPA. It is noteworthy that the amount of N–H in HB-DPPA is much lower than that of epoxy in DGEBA, leading to a quite blunt and weak peak in the thermogram.

### 2.3. Flammability

**Figure 2:**
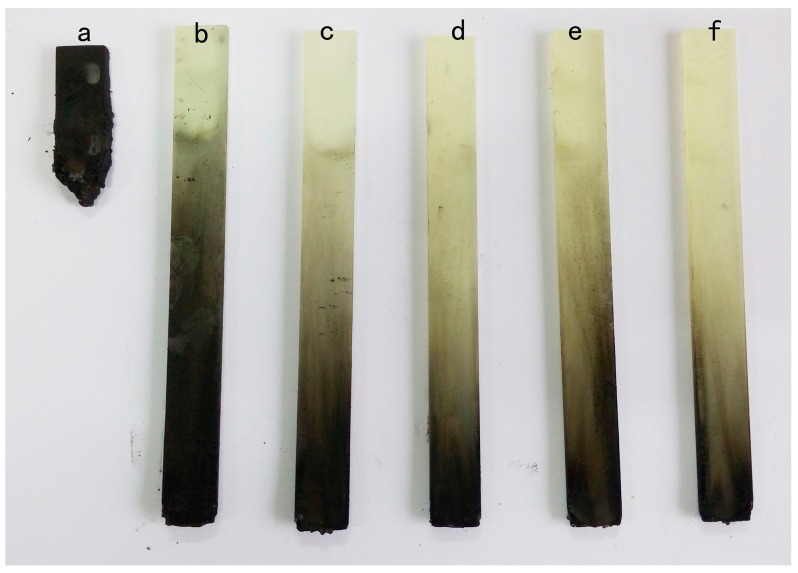
Digital photos of (**a**) EP-0; (**b**) EP-0.5; (**c**) EP-1; (**d**) EP-1.5; (**e**) EP-2; and (**f**) EP-2.5 samples after UL-94 test.

As is widely known, the limiting oxygen index (LOI) measurement and UL-94 vertical burning test are commonly used as indicators to evaluate the flammability characteristics of epoxy resin [32]. The results of LOI and UL-94 vertical burning tests are presented in Table 1. LOI increases gradually with a rise in HB-DPPA content, and reaches a high value of 29.3% for EP-2. At the same time, it is observed that severe combustion is followed by a quick diffusion of flame and intense burning drips from EP-0 owing to the high combustible nature of DGEBA/DDM. Interestingly, during the UL-94 vertical burning tests, the burning behavior of the cured epoxy changes greatly after the introduction of HB-DPPA, even a tiny amount. The dripping phenomenon is not observed anymore and the burning time decreases sharply. As shown from the digital photos (Figure 2), the cured epoxy resins with HB-DPPA have almost the same shape and dimension before and after UL-94 test. In regard to the fire safety requirement, 2.0 wt % of HB-DPPA (the corresponding phosphorus content of the system is 0.18 wt %) is needed to aid DGEBA/DDM for achieving the UL-94 V-0 rating. As for EP-2 and EP-2.5, a char layer is generated rapidly after ignition, which is then intumesced by the emission of gaseous products that consequently form airflow inside out as the result of pressurization. Eventually, the flame extinguishes right after the igniter is removed, and the smoke is completely blown out from the specimen. To the best of our knowledge, such low phosphorus content is hardly seen in this field, particularly for DGEBA/DDM system. Therefore, it is known that the usage of HB-DPPA provides DGEBA/DDM with super effective flame retardancy.

A cone calorimeter is an effective tool to evaluate the forced combustion behavior of polymer materials [31,33]. The main combustion parameters of EP-0 and EP-2 offered by cone calorimeter measurements are shown in Table 2.

Clearly, the TTI value increases from 54 s of EP-0 to 65 s of EP-2, implying that the accumulation rate of the combustible volatiles on the surface of the flame-retarded material decreases; thus a longer time is needed to reach the critical concentration for ignition. Compared with EP-0, the peak HRR, mean HRR, and THR values of EP-2 decrease by 9.1%, 8.7%, and 13.4%, respectively, while the residual weight significantly increases by 80%. The HB-DPPA molecule contains both P–C and P=O bonds that might cleave in advance and subsequently forms pyrophosphate or polyphosphate substances, being able to promote the dehydration of the polymer matrix and the formation of a char layer [32]. The reduction in mean HRR and mean MLR indicates that the flame retardation slows down combustion of the polymer matrix. In correlation with the drastic increases of residual weight, the stable char layer is proven to be formed during the combustion, which protects the underlying material from heat exchange and gas release. Therefore, it can be preliminarily concluded that HB-DPPA exerts a flame retardancy effect on the condensed phase.

### 2.4. Thermal Analysis

**Figure 3:**
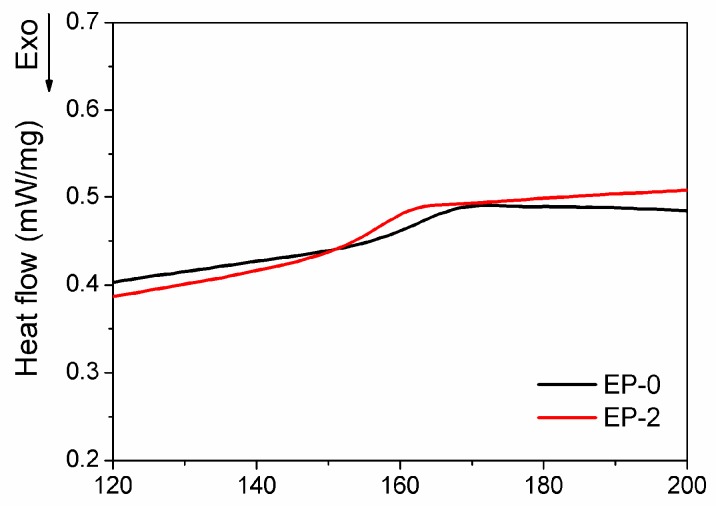
Differential scanning calorimeter (DSC) thermograms of the cured epoxy.

The glass transition temperature (T_g_) of cured epoxy was evaluated from DSC thermograms in Figure 3. A slight decrement on T_g_ emerges following the flame retardation, in which the value decreases from 162.2 °C of EP-0 to 157.4 °C of EP-2. The result is caused by the reduction of crosslink density of the thermoset that bears the bulky rigid aromatic group of the HB-DPPA molecule [23,26]. 

**Figure 4:**
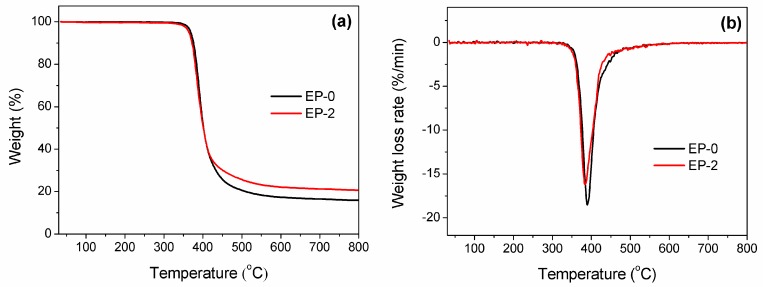
(**a**) TGA and (**b**) DTG curves of the cured epoxy.

To investigate the impact of HB-DPPA on the thermal stability and decomposition of DGEBA/DDM, Thermogravimetric analysis (TGA) was carried out at a constant heating rate of 10 °C/min under a nitrogen atmosphere. According to the results shown in Figure 4 and Table 3, it is known that both cured epoxies possess single-step decomposition from the cleavage of ether linkage and alkyl chain [34], though the T_5%_ and T_max_ of EP-2 are slightly lower than those of EP-0 due to the presence of phosphorus-containing groups that decompose at a relatively lower temperature [35].

In fact, the thermal stability of epoxy thermoset mostly decreases after the flame-retarded modification by organophosphorus compounds. The introduction of DOPO derivatives drops T_5%_ by 25–30 °C for the flame-retardant DGEBA/DDM [36,37]. As in our previous study using DPPA, the reduction of T_5%_ is adequately suppressed but still over 10 °C [26]. In addition to the superior efficiency in the flame-retardant aspect, where a smaller amount of HB-DPPA is needed for passing UL-94 test, the inclusion of an aromatic benzene ring in the HB-DPPA molecule provides a more rigid structure to the polymer network. As a result, the thermal stability of the flame-retardant epoxy maintains a high level, in which the value of T_5%_ only decreases by 4.6 °C. This feature makes the flame-retardant epoxy suitable for the high-temperature soldering process of printed circuit boards (PCBs), which commonly ranges from 350 °C to 380 °C. It is noteworthy that the decrement in mass loss rate signifies a deceleration of the combustible volatiles emission, which agrees with the extension of TTI in the cone calorimeter test.

**Figure 5:**
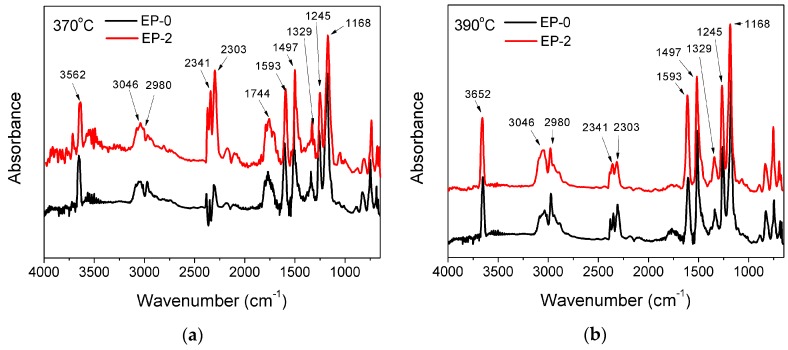
TGA–FTIR curves of EP-0 and EP-2 at (**a**) 370 °C and (**b**) 390 °C.

The gaseous product from the thermal degradation of cured epoxy was characterized using the TGA–FTIR method. Based on the TGA result, EP-0 and EP-2 both exhibit singular decomposition process. Accordingly, the FTIR spectra at the initial and maximum degradation temperatures (corresponding to 370 and 390 °C, respectively) are illustrated in Figure 5. The major decomposition products of DGEBA/DDM are observed from the following peaks on the EP-0 spectrum: 3500–3800 cm^−1^ (phenol derivatives, water, or amine compounds), 1593, 1515, 1497, and 1329 cm^−1^ (aromatic compounds), 3046 and 2980 cm^−1^ (aliphatic hydrocarbons), 1744, 1245, and 1168 cm^−1^ (ester and ether compounds), 2341 and 2303 cm^−1^ (CO_2_), and 2187 and 2116 cm^−1^ (CO) [38,39]. Epoxy thermoset flame-retarded by phosphorus-containing compounds commonly generates phosphorus-containing volatiles at high temperature, which can be distinctly analyzed from the TGA–FTIR spectrum. Intriguingly, the spectra of EP-2 are extremely similar to those of EP-0, in which almost no additional peak is detected at both temperatures despite the introduction of HB-DPPA. It indicates that unlike the common organophosphorus compounds, such as DOPO and its derivatives, HB-DPPA chemically bonded to epoxy does not experience further decomposition to generate volatilizable phosphorus-containing species, which might be caused by the greater stability of the phosphorus element attached with two benzene rings. The result suggests that HB-DPPA mainly exerts its flame-retardant effect through improvement of the condensed phase, while the impact on the gaseous phase seems to be insignificant.

**Figure 6:**
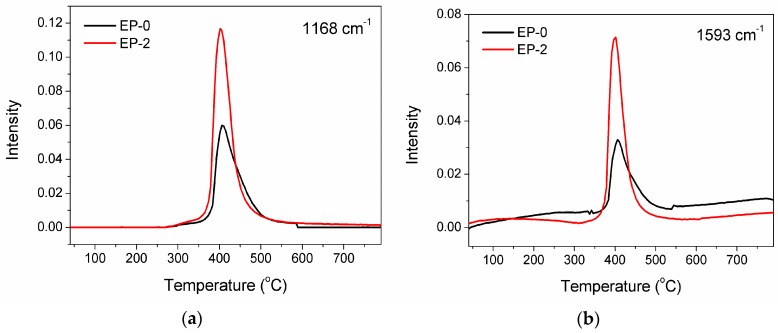
Absorbance intensities of (**a**) ether (1168 cm^−1^) and (**b**) aromatic compounds (1593 cm^−1^) emitted from decomposition of EP-0 and EP-2.

However, there exists an alteration in the absorbance intensity of pyrolysis products. Two main degradation products of DGEBA/DDM, ether and aromatic compounds (the corresponding characteristic peaks appear at 1168 and 1593 cm^−1^, respectively), are selected to be studied further, and the quantitative results of absorbance intensity are shown in Figure 6. For the equal sample weight, the detected signals for EP-2 are much stronger than those for EP-0. The result signifies the stimulating degradation effect of the phosphorus-containing moiety in HB-DPPA, which promotes decomposition and causes excessive emission of volatile substances as the gas source to induce the blowing-out effect.

**Figure 7:**
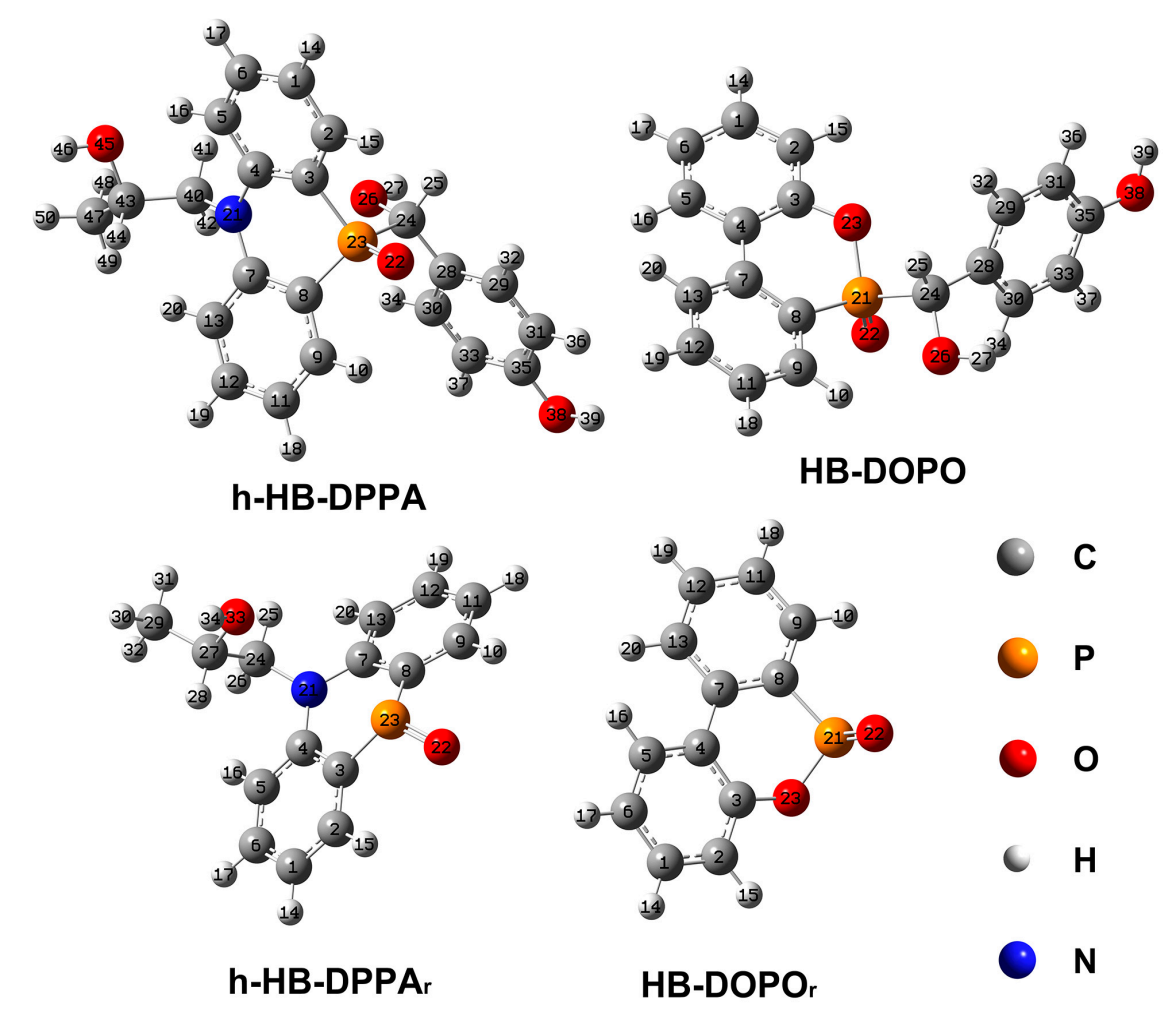
Optimized molecular structure of HB-DPPA and HB-DOPO moieties.

To clarify the higher stability of the phosphorus-containing group in HB-DPPA, the molecular structures of the related moieties are calculated by DFT/B3LYP/6-31G(d) level with Gaussian 09 software package (Figure 7), along with the length of covalent bonds adjacent to the phosphorus atom (Table 4). There is a general knowledge that the shorter the bond length, the higher the energy needed to dissociate the bond. In this work, bond length is calculated to describe the stability of the chemical bond. For EP-2, N–H in HB-DPPA reacts with the epoxy group in DGEBA. To simplify the calculation, such reaction product moiety adjacent with the nitrogen atom may be replaced with 2-hydroxypropyl, named h-HB-DPPA. For comparison, DOPO reacting with 4-hydroxybenzaldehyde is introduced and named HB-DOPO. It has been verified that the simplified calculation does not affect the bond length result in such a moiety. The result proves that the P–C bond in both structures (P23–C24 in h-HB-DPPA and P21–C24 in HB-DOPO) possesses the largest length, therefore such a P–C bond in the cured epoxy networks might break first at the high temperature producing radicals h-HB-DPPA_r_ and HB-DOPO_r_.

P–C bonds in h-HB-DPPA_r_ (P23–C3 and P23–C8) are shorter than those in HB-DOPO_r_ (P21–C8), and the former possess greater stability that might restrain further decomposition to generate phosphorus-containing species (PO• and PO_2_•). In fact, cleavage of the covalent bonds may also be influenced by the overall structure of the polymer network. Although further study is needed to show how the complex molecule affects the decomposition process, HB-DPPA might not decompose into the volatilizable phosphorus-containing species, as seen from our experimental results.

### 2.5. Char Layer

**Figure 8:**
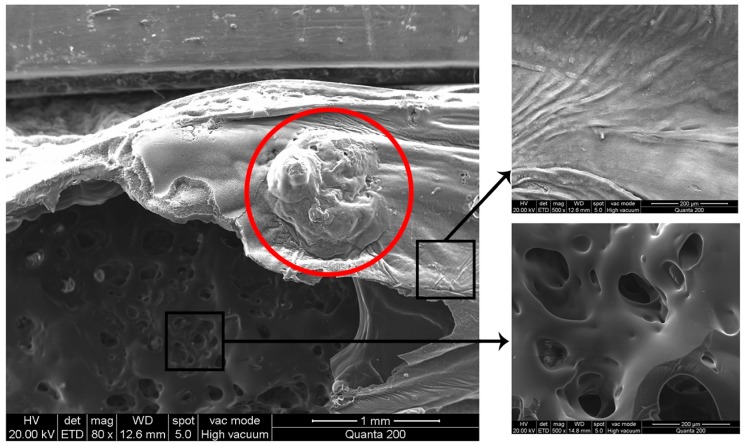
Scanning electron microscopy (SEM) images of the char residue of EP-2 after vertical burning test.

The flame-retardant mechanism of HB-DPPA in the condensed phase can be further studied from the char layer. The morphology of the char residue collected from the EP-2 sample after the vertical burning test is investigated by scanning electron microscopy (SEM) (Figure 8). It can be clearly seen that the char residue has a compact external surface with a multiporous structure inside. The compact layer acts as a barrier to protect the unburned matrix from the attack of heat and oxygen. A convex expanded layer is observed and marked with a circle. According to the visualization result, the rigid char layer is formed during combustion, while the evolved gases accumulate in the cavity to expand the char layer. Furthermore, due to the increase in the inner gas pressure following the thermal decomposition of the polymer matrix, the char layer is subsequently broken and the gases gush out of the site. The spurted airflow with high rate is able to blow the flame away from the material surface, thus realizing the blowing-out effect.

**Figure 9:**
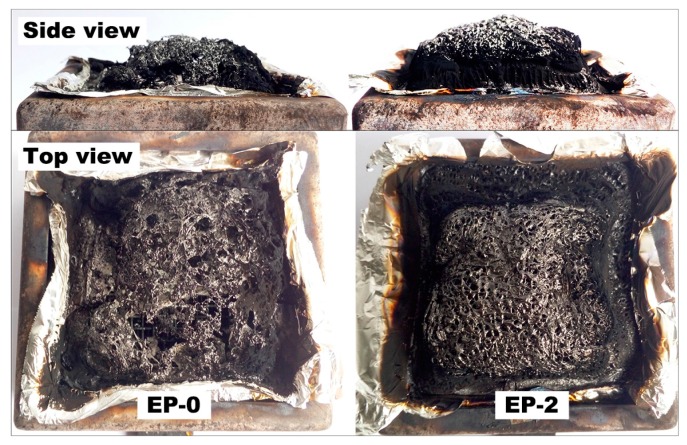
Digital photos of the char residue of EP-0 and EP-2 after cone calorimeter test.

**Figure 10:**
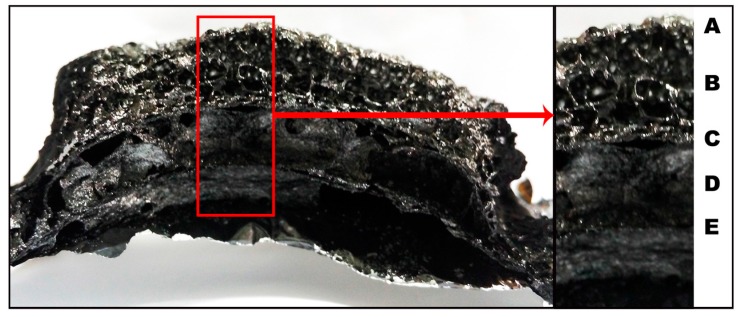
Digital photo of the interior part of EP-2 residual char after cone calorimeter test.

The char layers of EP-0 and EP-2 are also examined after the cone calorimeter test (Figure 9). The residual char of EP-0 seems to be quite thin, loose, and fragmentary, with large holes on the top surface. This is indicative of a lack of strength in the char layer formed during combustion, which suffers severe breakage by the heat and gas diffusions. As for EP-2, an expanded bulky char layer with compact and firm top surface is observed. In order to examine the interior characteristics, the char is cleaved vertically in the middle and the side view of the interior part is exhibited in Figure 10. There exists a unique char structure with five discernible layers, each with different morphologies and features.

**Figure 11:**
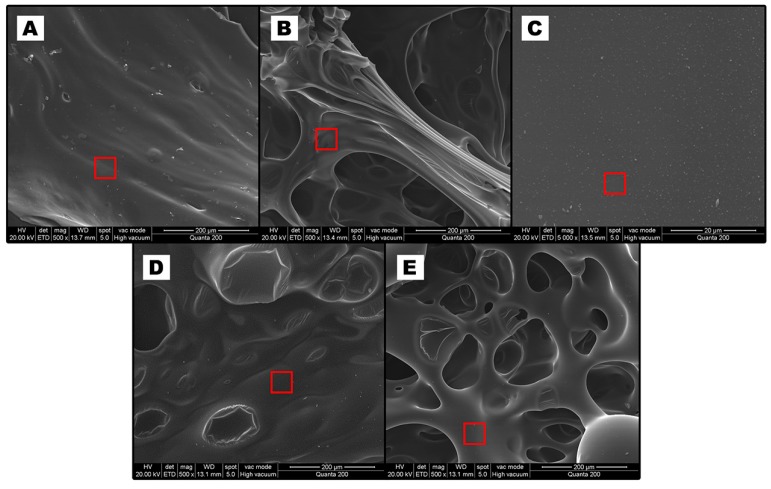
SEM images of different layer in the char residue of EP-2 after cone calorimeter test. The red squares indicate the scanning areas of EDS measurement.

The morphology of the layered char is further analyzed by SEM (Figure 11) and the elemental composition is obtained from the area indicated by the red square in the figures using energy dispersive spectroscope (EDS) (Table 5). The layers A and C show continuous and sealed morphology with higher phosphorus content. The layer B demonstrates an open-cavity structure that supports the above layer. Convex spheres are clearly evidenced from layer D, determining the presence of lower pressure gases. The innermost layer E presents multiporous morphology with a smaller size of hole as compared with that in layer B. Moreover, there are only a few phosphorus and nitrogen atoms in the layers exhibiting open-cavity structure (B and E), suggesting that the phosphorus-containing substances mostly migrate upwards and accumulate to form phosphorus-rich carbonaceous layers (corresponding to layers A and C). The scantiness of nitrogen indicates the stimulating degradation effect of the phosphorus-containing substances, which accelerates the decomposition of the thermoset and the generation of outward gases.

**Figure 12:**
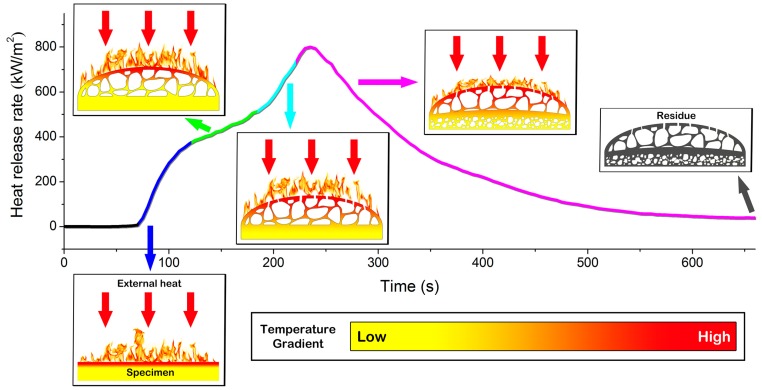
Formation mechanism of the unique char.

According to the above discussion, the formation mechanism of the unique char layer is proposed in Figure 12, along with the HRR curve to add the time scale and relate it to the combustion circumstance. Firstly, the sample is ignited from the irradiation of external heat and starts to decompose, which is represented by the release of heat (blue line). The decomposition of the phosphorus-containing moiety in the upper surface promotes the dehydration of the epoxy matrix, thus rapidly generating compact char layer A. The pyrolysis gases from the substance underneath cause the expansion of the char layer and the open-cavity structure layer B is formed accordingly. As a result of the formation of these two layers that act as a heat barrier, the heat release is decelerated in this period (green line). With an increase in the heat, the continually accumulated gases in layer B successfully break through the top layer A and gush outwards; meanwhile, the external heat infiltrates deeper and the lower layer subsequently decomposes, as manifested by the intensification of heat release (turquoise line). Moreover, another compact firm layer, C, is constructed. Since layer B possesses a cavity structure filled with air, the thermal conduction slackens and a heat gradient subsequently emerges, as reflected by the temperature distribution. The pyrolytic gases generated from the layers beneath (D and E) are no longer able to perforate the char layer above due to the lack of inner pressure. Therefore, they tend to construct a convex sphere in layer D. Following the exhaustion of combustion fuel from the decomposition of material, the heat release rate decreases (purple line) and the flame eventually extinguishes, leaving the unique residual char layer.

**Figure 13:**
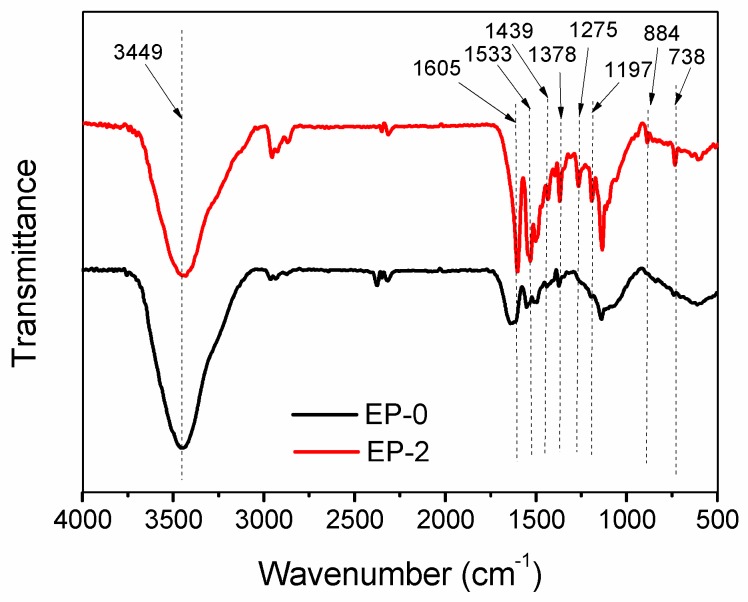
FTIR spectra of the char residue of EP-0 and EP-2 after cone calorimeter test.

The chemical structure of the char residue after the cone calorimeter test is further analyzed by FTIR spectroscopy (Figure 13). Unlike the gaseous phase, the utilization of HB-DPPA brings about a massive discrepancy in the FTIR spectrum of char residue, presuming that the chemical composition of the char residue is altered following the flame retardation. Compared with EP-0, several new peaks are detected in EP-2, which are attributed to the phosphorus-containing bands as follows: 1439 cm^−1^ (P–Ar), 1275 cm^−1^ (P=O), 1197 and 884 cm^−1^ (P–O–Ar), and 738 cm^−1^ (Ar–H of substituted aromatic ring of DPPA moiety) [40]. In addition, the intensification of peaks at 1605, 1533, and 1378 cm^−1^ signifies the formation of more aromatic carbon networks during the combustion. The spectroscopy result further implies that the phosphorus-containing substances are left in the condensed phase and join in the formation of a phosphorus-rich char layer, in addition to the stimulation of carbonization of the polymer matrix.

## 3. Experimental Section

### 3.1. Materials

Epoxy (DGEBA, epoxy value = 0.51 mol/100 g) was provided by SINOPEC Assets Management Corporation Baling Petrochemical Branch, Beijing, China. 4,4′-Methylenedianiline (DDM) was supplied by Shanghai Titan Scientific Co., Ltd., Shanghai, China. DPPA was synthesized in the laboratory according to our previous work [26]. 4-Hydroxybenzaldehyde (HB) was purchased from Shanghai Aladdin Reagents Co., Ltd., Shanghai, China.

### 3.2. Synthesis of HB-DPPA

**Scheme 1:**
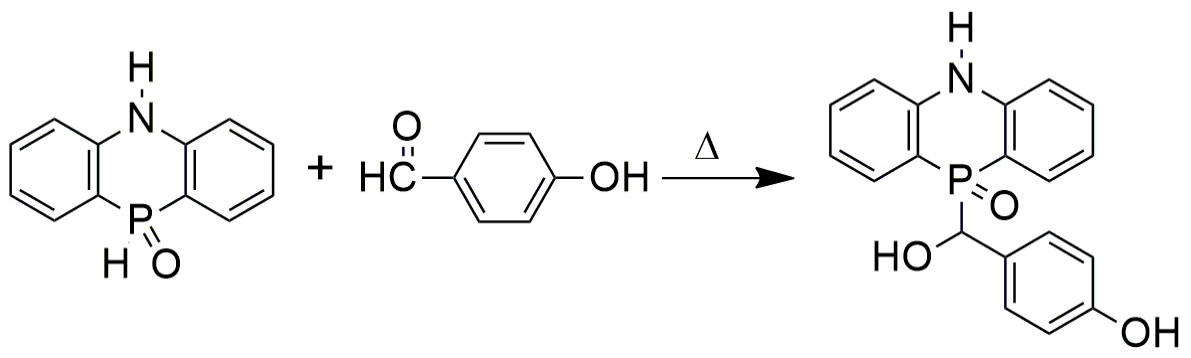
Synthesis route of HB-DPPA.

The synthesis route of HB-DPPA is shown in Scheme 1. Typically, DPPA (21.5 g, 0.10 mol), HB (13.4 g, 0.11 mol), and ethanol (200 mL) were introduced into a 500-mL round-bottomed three-neck glass flask equipped with a mechanical stirrer and reflux condenser. The mixture was heated to 78 °C and stirred until DPPA and HB were dissolved completely. Then, the reaction lasted for another 5 h. Having been cooled to room temperature, the filtered precipitate was washed with dichloromethane and dried in vacuo at 40 °C. Finally, a white powder was obtained (yield = 95%).

### 3.3. Preparation of Cured Epoxy

DGEBA was thermally co-cured with various weights of DDM and HB-DPPA. The stoichiometric formulation for the flame-retarded epoxy at different phosphorus contents is listed in Table 6, where the sum of the reactive protons is equal to that of the epoxy groups.

Typically, DGEBA was added into a three-necked flask and heated to 180 °C in an oil bath. HB-DPPA was then added slowly to the resin and mixed under stirring until the additive was completely dissolved and the mixture became clear. Having been cooled down to 80 °C, DDM was added into the mixture and stirred until the mixture was clear again. Then, the mixture was poured into a preheated mold and degassed in a vacuum oven at 80 °C for 30 min. Finally, the mixture was cured in the following steps: 80 °C for 1 h, 150 °C for 2 h, 180 °C for 2 h, and 200 °C for 2 h.

### 3.4. Characterization

FTIR spectra from 400 to 4000 cm^−1^ were recorded by a Vertex70 spectrometer (Bruker, Billerica, MA, USA). ^1^H nuclear magnetic resonance (NMR) spectra were obtained on a DRX400 spectrometer (Bruker) using DMSO-*d*_6_ as solvent. Mass spectrometry (MS) results were obtained from a maXis impact mass spectrometer (Bruker). DSC analysis was conducted on a DSC-200F3A01 thermal analyzer (Netzsch, Selb, Germany). The glass transition temperature (T_g_) was measured by DSC at a heating rate of 10 °C/min under a nitrogen atmosphere; the sample was annealed in advance to eliminate thermal history; and the middle of the incline was defined as T_g_. The LOI was measured by an oxygen index instrument (Fire Testing Technology, East Grinstead, UK) according to ASTM D2863-97 with specimen dimensions of 150 × 6.5 × 3 mm^3^. The vertical burning UL-94 test was performed on a UL-94 flammability meter (Fire Testing Technology) according to ANSL UL 94-2009 with specimens’ dimensions of 130 × 13 × 3.2 mm^3^. The cone calorimeter result was obtained using a CONE-SC60 cone calorimeter (Fire Testing Technology) according to ISO 5660 under an external heat flux of 35 kW/m^2^. The sample size for the cone calorimetric analysis was 100 × 100 × 5 mm^3^. TGA was carried out on a TG-209F1 thermogravimetric analyzer (Netzsch) at a heating rate of 10 °C/min under a nitrogen atmosphere. TGA-FTIR measurements were performed using a STA449C/3MFC/G thermogravimetry–Fourier transform infrared spectrometer (Bruker) at a heating rate of 20 °C/min under nitrogen atmosphere. SEM observation was performed using a Nova Nano SEM430 (FEI, Eindhoven, The Netherlands) equipped with EDS. The samples’ surface was coated with a thin layer of gold–palladium alloy prior to the experiment.

## 4. Conclusions

HB-DPPA was successfully synthesized and its flame retardancy on DGEBA/DDM was found to be highly effective, as merely 0.18 wt % of the phosphorus content was sufficient to pass UL-94 V-0 rating. Such high flame retardancy was derived from the peculiar mechanism in the condensed phase due to the higher stability of phosphorus atom in HB-DPPA, which could not be decomposed into the volatilizable phosphorus-containing species. The unique char layer and blowing-out effect were brought about as a consequence of using such an exclusive molecular structure. In addition to the super-efficient flame retardancy, the initial decomposition temperature of the flame-retardant epoxy only decreased by 4.6 °C as compared with the unmodified version. Further computational investigation regarding the decomposition pathways of such flame-retardant molecules and thermoset systems could be performed in future work.

## Figures and Tables

**Table 1:** LOI and UL-94 test results of cured epoxy.

Sample ID	LOI (%)	*t* ^1^ (s)	Dripping	UL-94 Rating
EP-0	24.1	>180	Yes	No Rating
EP-0.5	28.2	36	No	No Rating
EP-1	28.3	32	No	No Rating
EP-1.5	28.8	25	No	V-1
EP-2	29.3	8	No	V-0
EP-2.5	29.5	5	No	V-0

^1^ *t*: total time of the first and second burning times for five specimens in UL-94 test; LOI = limiting oxygen index.

**Table 2:** Cone calorimeter data of EP-0 and EP-2.

Sample ID	TTI (s)	Peak HRR (kW/m^2^)	Mean HRR (kW/m^2^)	THR (MJ/m^2^)	Mean MLR (g/s)	Residual Weight (wt %)
EP-0	54	880	312	187	0.093	12.0
EP-2	65	800	285	162	0.081	21.6

TTI: time to ignition; HRR: heat release rate; THR: total heat release; MLR: mass loss rate.

**Table 3:** TGA data of EP-0 and EP-2.

Sample ID	T_5%_ (°C)	T_max_ (°C)	R_max_ (%/min)
EP-0	368.9	389.6	18.5
EP-2	364.3	383.7	16.2

T_5%_: 5% weight loss temperature; T_max_: maximum decomposition rate temperature; R_max_: maximum weight loss rate.

**Table 4:** Calculated bond lengths of HB-DPPA and HB-DOPO moieties.

Bonds	Bond Length (Å)		Bonds	Bond Length (Å)	
	h-HB-DPPA	h-HB-DPPA_r_		HB-DOPO	HB-DOPO_r_
P23–C3	1.796	1.798	P21–C8	1.808	1.816
P23–C8	1.796	1.797	P21–O23	1.654	1.668
P23=O22	1.503	1.516	P21=O22	1.489	1.500
P23–C24	1.881	-	P21–C24	1.863	-

**Table 5:** EDS results of the layered char of EP-2 after cone calorimeter test.

Weight Percent (wt %)	Char Layer				
	A	B	C	D	E
C	74.85	74.62	69.14	73.64	86.97
O	23.33	18.73	17.33	14.99	11.56
N	-	5.72	11.93	10.15	0.43
P	1.82	0.93	1.60	1.22	1.04

**Table 6:** Stoichiometric formulation of the epoxy system.

Sample ID ^1^	DGEBA (wt %)	DDM (wt %)	HB-DPPA (wt %)	*P* (wt %)
EP-0	79.83	20.17	0	0
EP-0.5	79.61	19.89	0.5	0.05
EP-1	79.39	19.61	1.0	0.09
EP-1.5	79.16	19.34	1.5	0.14
EP-2	78.94	19.06	2.0	0.18
EP-2.5	78.72	18.78	2.5	0.23

^1^ In the sample name EP-*X*, *X* represents the weight fraction of HB-DPPA in the DGEBA/DDM/HB-DPPA system.

## Supplementary Materials

The following are available online at www.mdpi.com/1996-1944/9/12/1008/s1. Figure S1: FTIR spectra of DPPA, HB and HB-DPPA, Figure S2: DSC thermograms of EP-0 and EP-2 at a heating rate of 5 °C/min, Table S1: curing parameters of EP-0 and EP-2, Figure S3: DSC thermogram of DGEBA/HB-DPPA at a heating rate of 5 °C/min.
