# Supplementary material for: Intrinsic Flame-Retardant and Thermally Stable Epoxy Endowed by a Highly Efficient, Multifunctional Curing Agent
Source: Materials (Basel). 2016 Dec 12;9(12):1008. doi: 10.3390/ma9121008 (PMC5456980; doi:10.3390/ma9121008)
Supplement: Supplementary file 1 [file materials-09-01008-s001.pdf]

Supplementary Materials: Intrinsic Flame-Retardant and Thermally Stable Epoxy Endowed by a Highly Efficient, Multifunctional Curing Agent

Chunlei Dong, Alvianto Wirasaputra, Qinqin Luo, Shumei Liu, Yanchao Yuan, Jianqing Zhao and Yi Fu

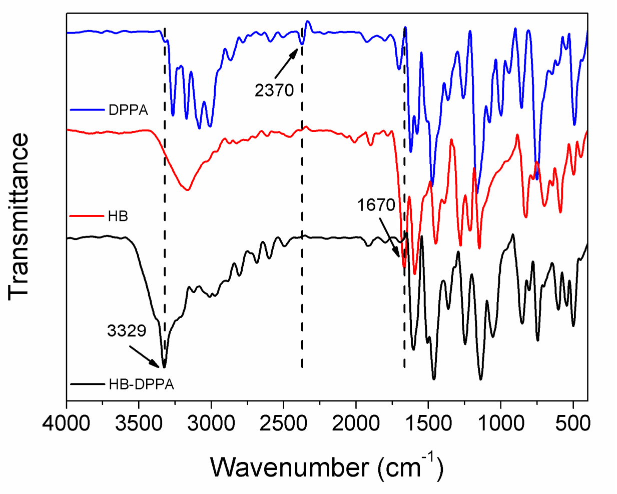

Figure S1. FTIR spectra of DPPA, HB, and HB-DPPA.

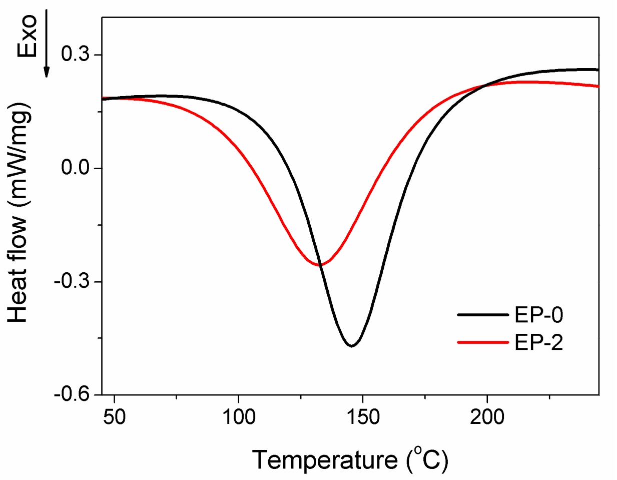

Figure S2. DSC thermograms of EP-0 and EP-2 at a heating rate of 5 °C/min.

Table S1. Curing parameters of EP-0 and EP-2.

Sample ID	T_o (°C)	T_p (°C)	T_e (°C)
EP-0	69.1	145.4	238.4
EP-2	54.5	132.1	215.7

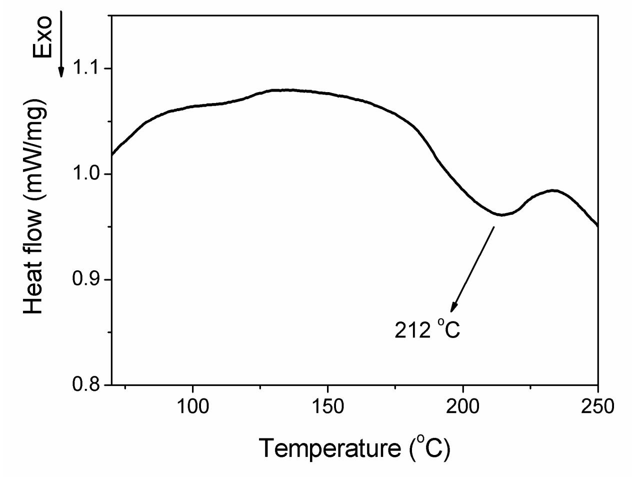

Figure S3. DSC thermogram of DGEBA/HB-DPPA at a heating rate of 5 °C/min.
